# Supplementary material for: Network growth models: A behavioural basis for attachment proportional to fitness
Source: Sci Rep. 2017 Feb 13;7:42431. doi: 10.1038/srep42431 (PMC5304319; doi:10.1038/srep42431)
Supplement: Supplementary Material [file srep42431-s1.pdf]

Network growth models: A behavioural basis for attachment proportional to fitness

Michael Bell*, Supun Perera, Mahendrarajah Piraveenan, Michiel Bliemer, Tanya Latty, Chris Reid

University of Sydney
Sydney, NSW 2008
Australia
*Corresponding author: michael.bell@sydney.edu.au

SUPPLEMENTARY MATERIAL

Network growth figures

For both homogeneous and tiered networks, we simulated network growth based on the attachment probabilities obtained through minimisation of maximum exposure to unfitness.

Fig. S1 shows a sample homogeneous network generated by our experiments, with number of nodes set to 1000 and number of links set to 1000. The node sizes represent fitness, and nodes are ordered by degree in a circle. We may observe that, in general, the higher the fitness, the higher the degree, even though, due to finite size effects in such small systems, resultant node degrees are sometimes not in the exact order of attachment probabilities.

Similarly, Fig. S2 shows a sample tiered network generated by our experiments, with the number of nodes set to 1000 organised into four tiers and number of links set to 1000. The nodes belong to four types in equal number and have log-normal fitness distributions with shape parameters σ_1=3, σ_2=1, σ_3=1, σ_4=0.1 representing suppliers, manufacturers, distributors and retailers respectively. The tiered structure is clearly visible. In Fig. S2, the node size is proportional to intrinsic fitness, while nodes from the four tiers are each arranged in a circle sorted by node degree. It could again be seen that nodes with higher fitness tend to have higher degrees, though this is more prominent in the supplier layer where the differences in fitness are more pronounced compared to the retailer layer where the differences in fitness are least pronounced. Though, as mentioned before, due to finite size effects the correlation between attachment probabilities and degrees is not perfect.

Note however that our work focusses on showing that the mechanism of minimising maximum unfitness results in attachment probabilities proportional to fitness, exactly matching fitness-based preferential attachment models. The network growth process that then results does not depend on how these probabilities were arrived at, and if we had used a preferential attachment mechanism such as LNFA (Log-Normal Fitness Attachment) to arrive at the attachment probabilities, the resulting networks would have been exactly the same. Therefore, the networks grown do not form part of our results, and are shown here only to complete the examples.

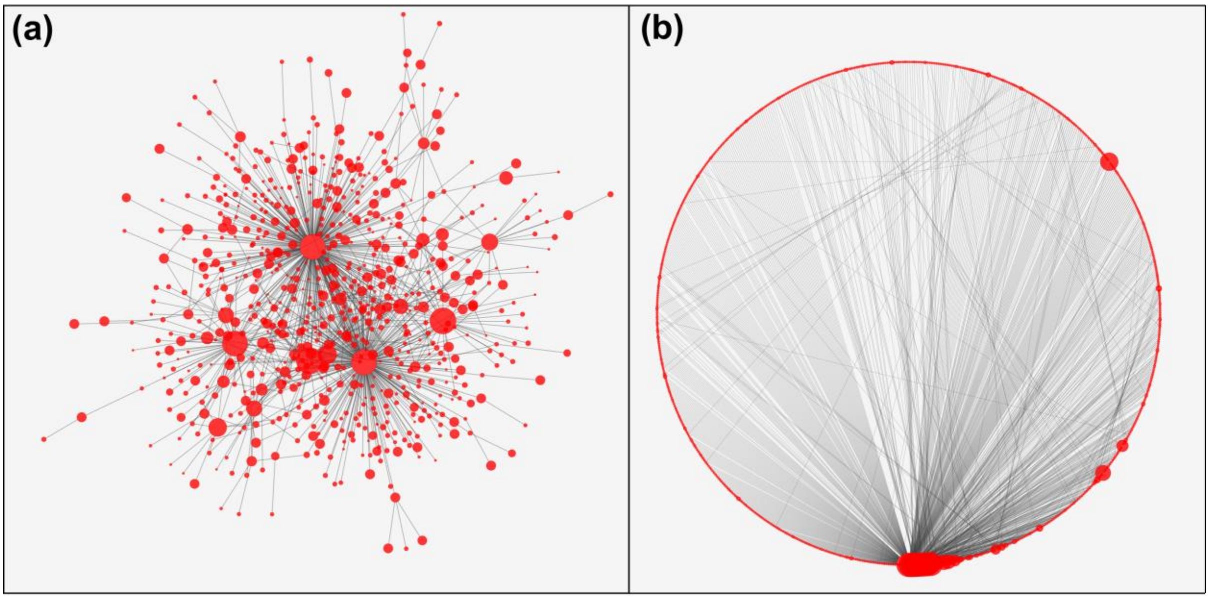

Figure S1: *The homogeneous network model, of size 1000 nodes. Note that any node can connect to any other node in this case – there are no constraints. (a) The network topology in force-directed layout, with nodes sizes proportional to fitness (b) Here, again the node size is proportional to intrinsic fitness, and all nodes are arranged in a circle according to node degree. We notice that in general, the larger nodes (the nodes with higher fitness) are also the nodes with the highest number of connections.*

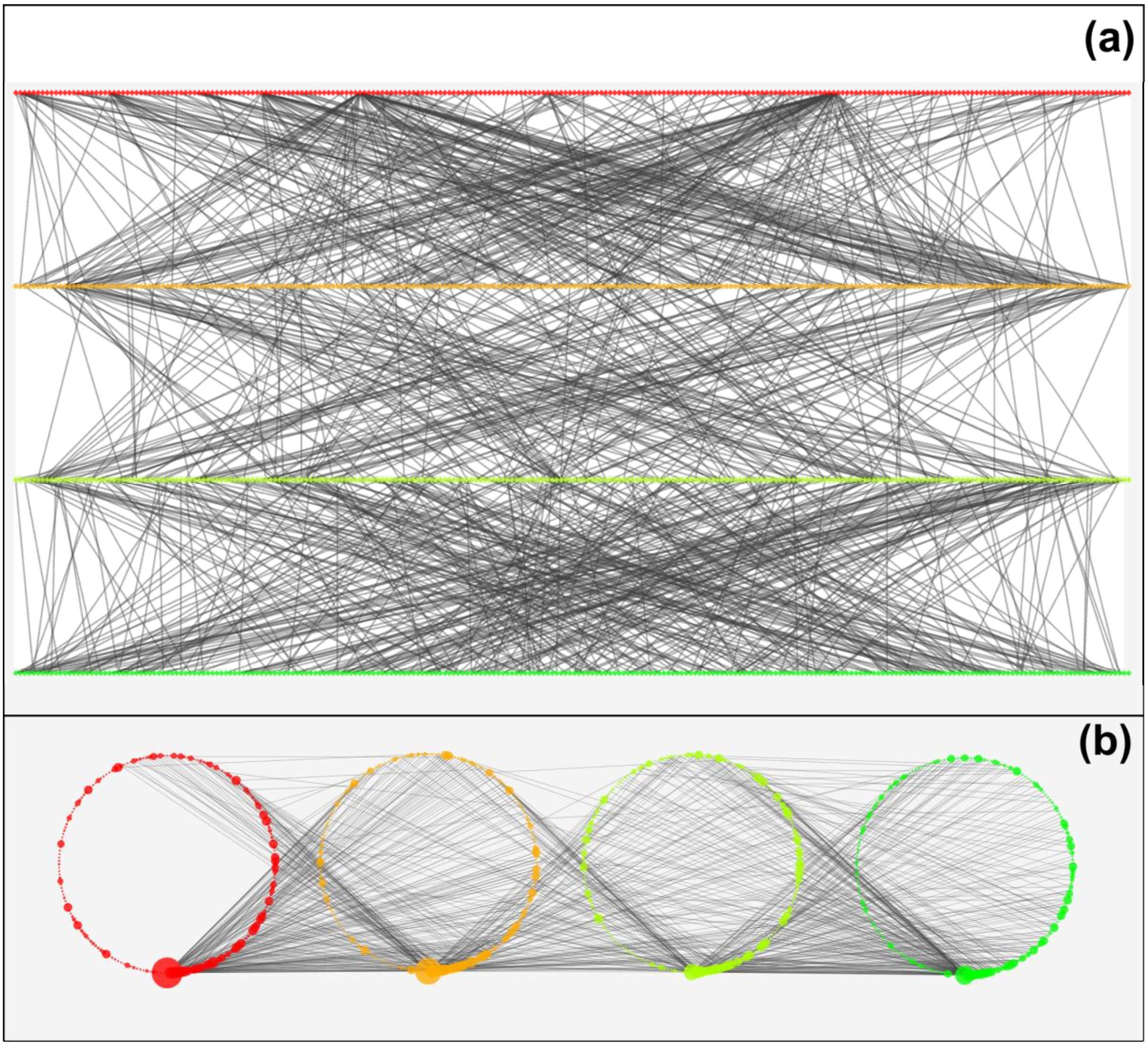

Figure S2: *The tiered network model, of size 1000 nodes. This example represents a supply chain, where the red, orange, light green and green nodes denote suppliers, manufacturers, distributors and retailers respectively. (a) The network topology. In the tiered network model, link formation is only allowed between adjacent tiers, and a hierarchical structure is evident. (b) Again, the node size is proportional to intrinsic fitness, and each type of node is arranged in a circle according to node degree. We notice that in general, the larger nodes (the nodes with high fitness) are also the nodes with the highest number of connections. This is most obvious in the supplier nodes where the fitness distribution has a high standard deviation, while least obvious in the retailer nodes where the fitness values are very similar between nodes.*
